# HEnRY: a DZIF LIMS tool for the collection and documentation of biomaterials in multicentre studies

**DOI:** 10.1186/s12859-020-03596-1

**Published:** 2020-07-08

**Authors:** Stephanie Heinen, Nick Schulze, Bernd Franke, Florian Klein, Clara Lehmann, Maria J. G. T. Vehreschild, Claas Gloistein, Melanie Stecher, Jörg Janne Vehreschild

**Affiliations:** 1 Faculty of Medicine and University Hospital Cologne, Department I for Internal Medicine, University of Cologne, Herderstr. 52-54, 50931 Cologne, Germany; 2 German Center for Infection Research (DZIF), partner site Bonn-Cologne, Cologne, Germany; 3 Institute of Virology, University Hospital Cologne, Fürst-Pückler-Str. 56, 50935 Cologne, Germany; 4 Medizinisches Versorgungszentrum, Fachbereich Infektiologie, University Hospital Cologne, Kerpener Str. 62, 50937 Cologne, Germany; 5 Head of Infectious Diseases, Department II of Internal Medicine, Goethe University Frankfurt, Frankfurt am Main, Germany; 6 Head of Clinical Microbiome Research Group, Department I for Internal Medicine, University of Cologne, Cologne, Germany; 7 Faculty of Medicine and University Hospital Cologne, Department II for Internal Medicine, University of Cologne, Cologne, Germany; 8 Department of Internal Medicine, Hematology/Oncology, Goethe University Frankfurt, Frankfurt am Main, Germany

**Keywords:** LIMS, Biobanking, Sample documentation, Sample storage, Sample management, Sample processing, DZIF, TP HIV, Multicentre studies, TI biobanking

## Abstract

**Background:**

Well-characterized biomaterials of high quality have great potential for acceleration and quality improvement in translational biomedical research. To improve accessibility of local sample collections, efforts have been made to create central biomaterial banks and catalogues. Available technical solutions for creating professional local sample catalogues and connecting them to central systems are cost intensive and/or technically complex to implement. Therefore, the Translational Thematic Unit HIV of the German Center for Infection Research (DZIF) developed a Laboratory Information and Management System (LIMS) called HIV Engaged Research Technology (HEnRY) for implementation into the Translational Platform HIV (TP-HIV) at the DZIF and other research networks.

**Results:**

HEnRY is developed at the University Hospital of Cologne. It is an advanced LIMS to manage processing and storage of samples and aliquots of different sample types. Features include:

- monitoring of stored samples and associated information
- data selection via query tools or Structured Query Language (SQL)
- preparation of summary documents, including scannable search lists
- centralized management of the practical laboratory part of multicentre studies (e.g. import of drawing schemes and sample processing steps),
- preparation of aliquot shipments, including associated documents to be added to shipments
- unique and secure identification of aliquots through use of customizable Quick Response (QR) code labels directly from HEnRY
- support of aliquot data transmission to central registries.

In summary, HEnRY offers all features necessary for a LIMS software. In addition, the structure of HEnRY provides sufficient flexibility to allow the implementation in other research areas.

**Conclusion:**

HEnRY is a free biobanking tool published under the MIT license. While it was developed to support HIV research in Germany, the feature set and language options, allow much broader applications and make this a powerful free research tool.

## Background

A biobank can be described as a biomaterial repository, in which biological samples and data of their respective donors are stored for use in biomedical research. It involves the systematic and standardized procurement, processing, annotation, storage, and ultimately distribution to the involved researchers [1]. Biobanks enable cross-purpose research studies by rapidly providing sufficient and well-described material for experimental and translational experiments. Linkage of biomaterial samples with clinical data provides important insights to phenotypic disease information that is crucial in the current research environment [2, 3].

Today, indexing, labelling, and storage of samples in professional biobanks is usually handled by Laboratory Information Management Systems (LIMS) that support the user with administrative and coordinative tasks of sample processing as well as the collection and analysis of identified analytical data [4, 5]. Ideally, LIMS also offer data exchange with other systems via interfaces. Many LIMS on the market offer flexible systems such as combinable modules, which can be added according to the customer’s needs.

The features of a LIMS have changed over the years from a basic sample tracking to a resource planning tool that manages multiple aspects of laboratory informatics. Common features of LIMS are workflow management, record keeping, inventory management, data exchange interfaces and reporting. They bring accuracy and accessibility to the flow of samples and laboratory data [6–9]. One particularly important feature is the possibility for automation to increase processing speed while reducing workload. These features are most effective for repetitive and predictable tasks [8, 10]. By standardizing documentation and providing SOPs for sample processing, a LIMS can improve data and sample quality and reliability by minimizing of documentation errors [1, 11–14].

By using a LIMS with a database accessible to each employee, individually managed spreadsheets can be replaced. Representation of the collected and complex data is simplified by the use of database queries. Regular backups can ensure the safety and availability of laboratory data.

Most LIM systems on the market have high initial costs or license fees. Small or newly founded working groups usually lack the financial means to purchase such a system [1]. For the specific purpose of collecting and processing samples of the Translational Platform HIV (TP-HIV) within the German Centre for Infection Research, we developed and here present the HIV Engaged Research Technology (HEnRY). Due to excellent user feedback and since we noticed that many of HEnRY’s features are required by a broad range of researchers, it was decided to put the tool under open license and make it available to the community.

## Implementation

### Development

HEnRY is released under the MIT licence [15]. The client is under development since 2014 using C# [16] with Windows Presentation Foundation (WPF) [17] and the. NET 4.5 [18–20] framework by an agile structured team. An installer package for Windows platforms (Windows 7 up to Windows 10), as well as the source code are available upon request. The manual and short instructions for the program are available on the website www.tp-hiv.de. A first production version was made available for participants of the TP-HIV on the February 15th, 2017.

All data is stored in a Microsoft SQL (MSSQL) database on a MSSQL Server 2012 or greater [21]. It is recommended to install the server and the databases in a protected network suitable for highly detailed and potentially stigmatizing patient data with considerable potential for misuse. For additional information, see Supplementary material 1, Supplementary material 2 and Supplementary material 3.

During the developmental process of HEnRY, a close collaboration with the laboratory staff, which represents the primary target group of the tool, and study coordinators was established. Both user groups gave continuous feedback to developed and requested new features, which were tailored to their needs.

User feedback in the form of meetings and questionnaires was obtained at regular intervals. Requests and bugs were fixed in the context of monthly updates. Additionally, the lead developer accompanied the lab employees in their daily work routine with HEnRY to streamline the program to the actual workflow in the lab.

The HEnRY LIMS software is in use at the University of Cologne since 2016, with currently seven active working groups.

### Workflow

**Fig. 1:**
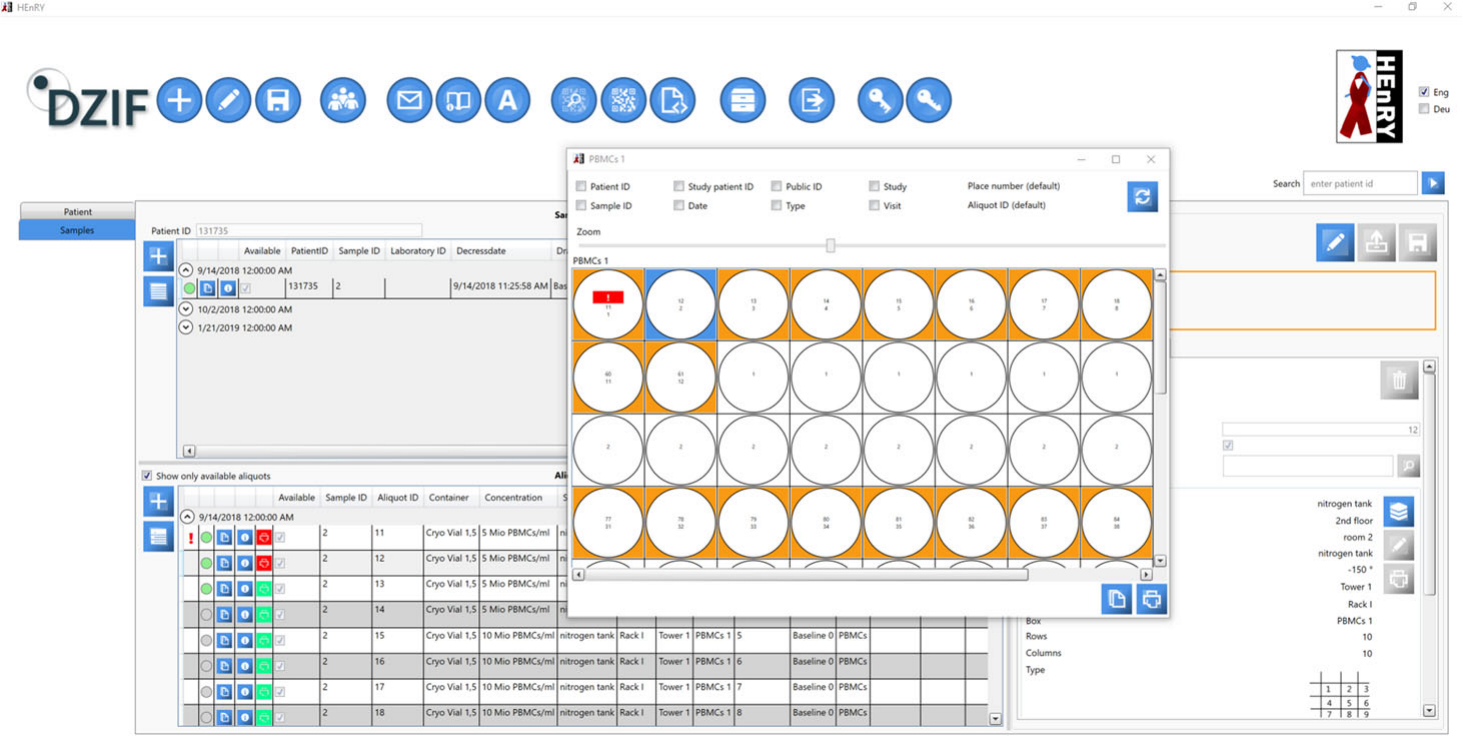
Snapshot of the bio banking view. The quick view option of the box is opened

HEnRY specializes in the documentation of the storage and processing of blood samples and aliquots in analytically working laboratories. It simplifies and accelerates laboratory work, increases the quality of documentation and minimizes potential sources of errors. Information about studies, patients, samples, aliquots and processing steps is stored in a structured manner (see Fig. 1). This information can be exchanged via pseudonym export and import functions, which improves cooperation between different participants of the studies. Additional information can be found in Supplementary material 4, Supplementary material 5 and Supplementary material 6.

**Fig. 2:**
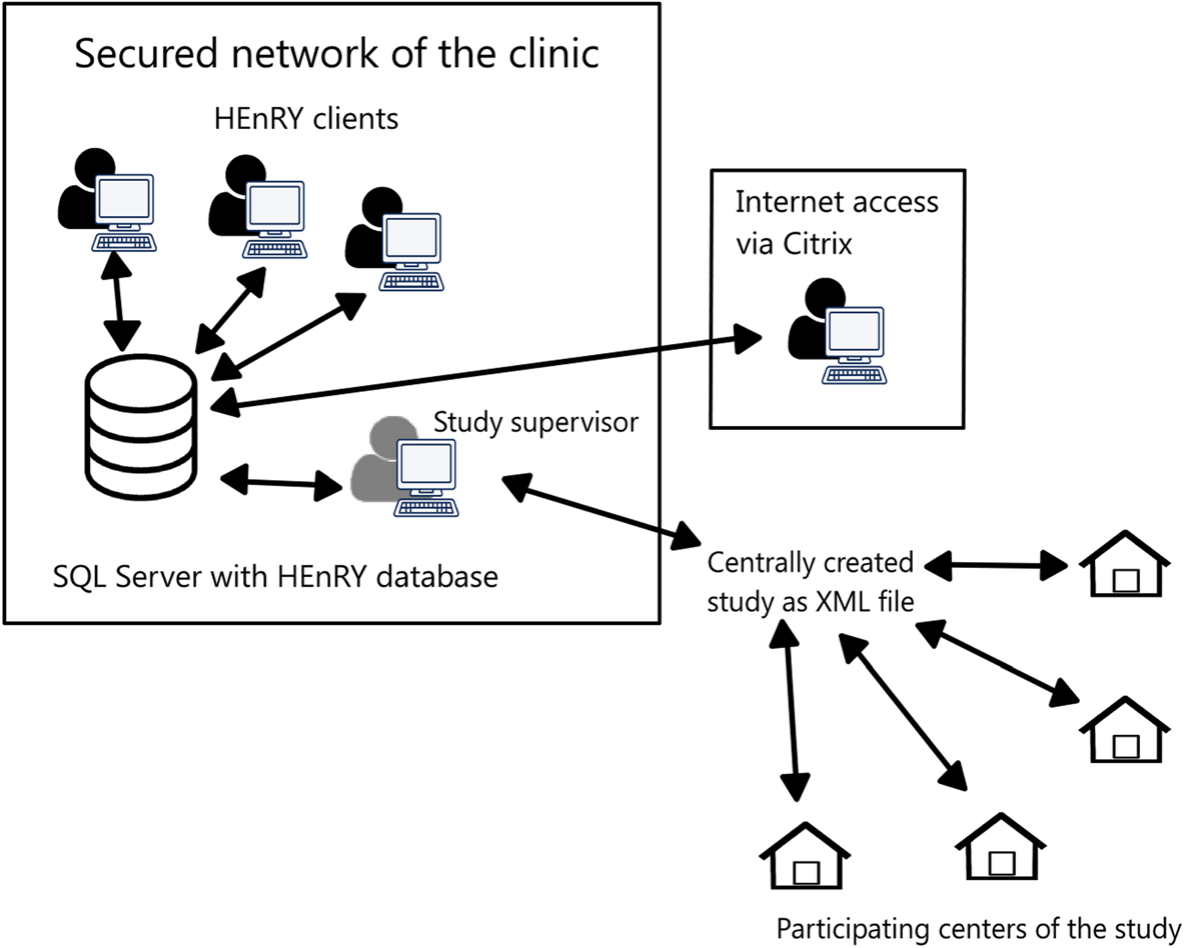
Scheme of HEnRY-usage and distribution of practical study data to participating centers in multicenter studies

Figure 2 shows a diagram of the workflow for multicenter studies and the use of HEnRY. An unlimited amount of users per study site is supported. Online access is available using the sites own Citrix access [22]. A study supervisor or study coordinator can develop the study properties, drawing schemes and processing steps and send the study design as an XML file to other participating centers.

In a setting of limited resources, HEnRY can be used as a standalone LIMS on one computer with e.g. SQL Server Express editions for structured documentation of biomaterial and self-adhesive label creation. Via the transfer of XML files, data for biomaterial can be shared with other laboratories, clinics or project partners.

### Study management

**Fig. 3:**
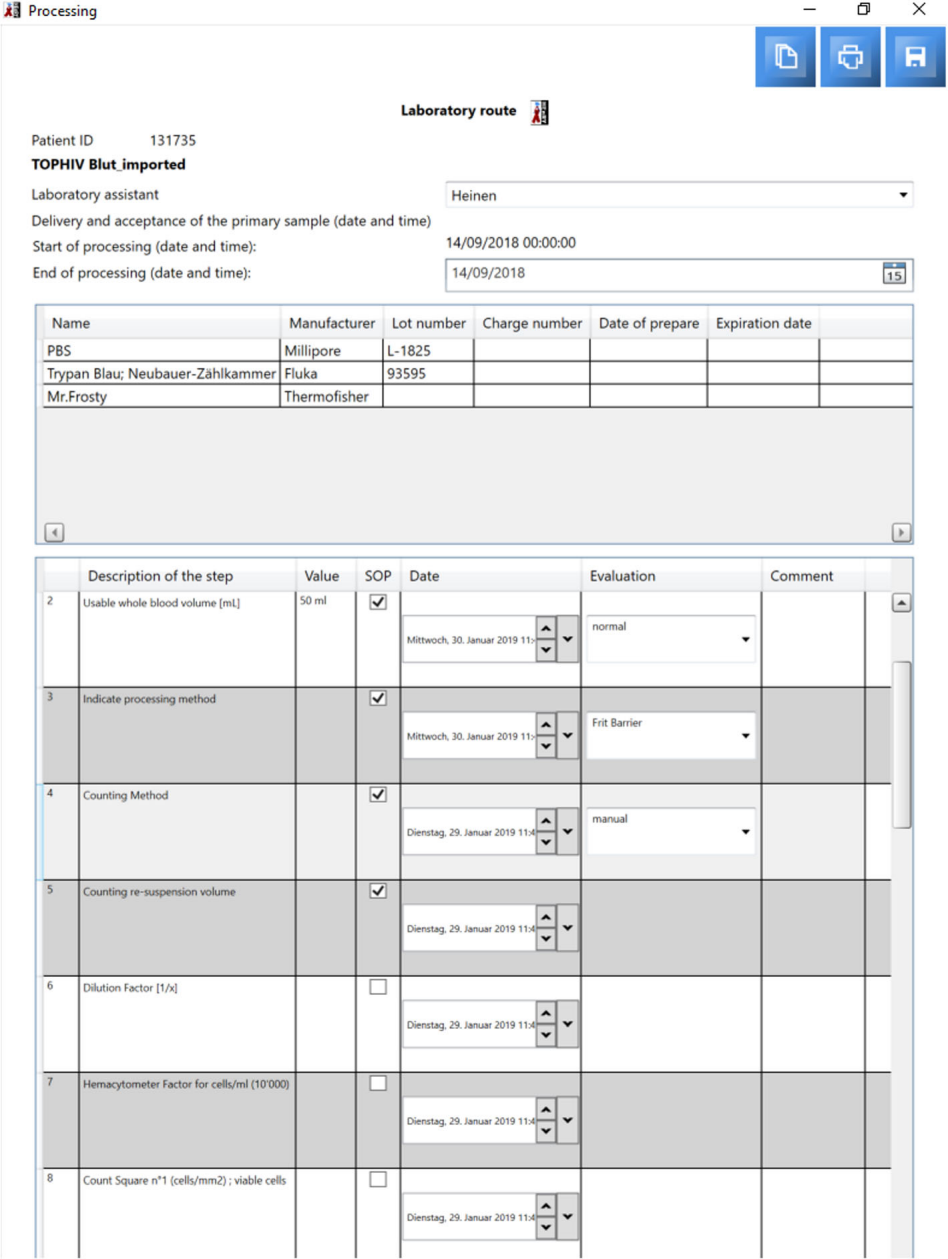
The processing letter in HEnRY used in the laboratory for the documentation of sample processing

**Fig. 4:**
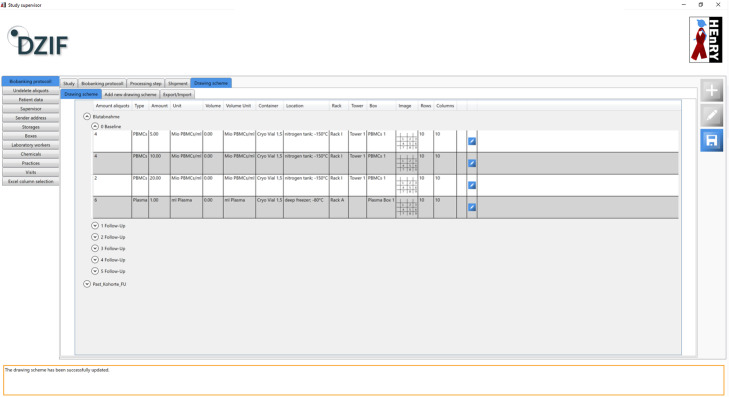
Snapshot of a drawing scheme in HEnRY

HEnRY offers the opportunity to manage any number of studies and respective workflows to support practical work in the laboratory. The name of the study, start and end date, contact persons, and whole laboratory books, down to descriptions of the chemicals to use, can be added to a study (see Fig. 3). More importantly, a scheme for blood draws, samples, and aliquots can be created for each study visit, allowing rapid processing of incoming samples. Information about the storage location can also be stored in the study scheme (see Fig. 4). A study scheme contains all information for samples after processing. E.g., if after processing 50 aliquots are derived from one sample, all information about creation date, aliquot type, amount, volume, container, study and location are the same for the 50 aliquots. By applying a study scheme, 50 aliquots with all properties are created in one click. Aliquots created via a study scheme are directly linked to the selected study. Different processing steps, including the used chemicals, are stored with the sample, if the processing has previously been assigned to the study (see Fig. 4).

**Fig. 5:**
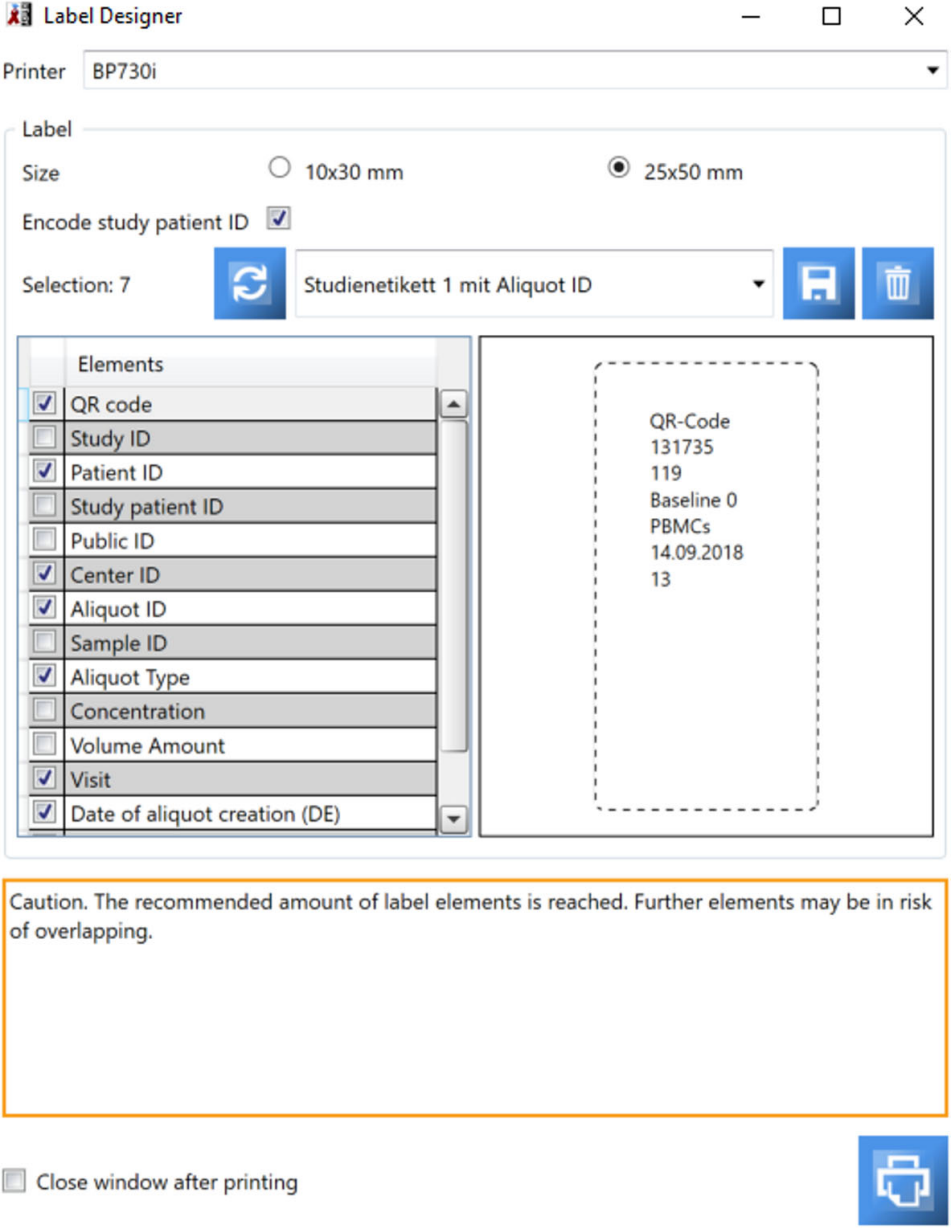
Label designer for the creation of customize labels

### Multicentre studies

Study schemes with all the practical information (visits with respective sampling procedures, processing steps, chemicals to use, addresses, contact persons, and more) can be exported to XML files [23]. A study coordinator can centrally design a study in HEnRY and distribute the XML file to all participating centers. For imported studies, all study specific fields are read-only and cannot be changed by the participating center. These fields are predefined by the protocol of the study (e.g. way of shipment for aliquots, addresses of participating centers, processing steps, and visit plans with respective sampling schemes). Only information specific to the local center, e.g. storage location, can be added to the imported drawing scheme.

This procedure was initially developed for the TopHIV study, which is centrally coordinated from working groups in Cologne and Hannover. The XML files for the study are available for download on the website of the TP-HIV [24].

### Backend

HEnRY offers the opportunity to work with different databases. The user chooses the database to work with when starting the program. Samples processed for different working groups or studies can be stored in separate databases to expedite analysis and restrict accessibility of the data.

### Storage management and documentation

Patient records can be created directly in HEnRY or linked to another clinical database. One such interface is available for the HIV Observation Software (HIObS) of the German Robert Koch-Institute, Berlin [25]. For data security reasons, HEnRY allows only pseudonymous patient records. Any number of IDs used in different studies can be stored as aliases for one patient record.

HEnRY offers a large range of properties, which can be documented for each sample or aliquot. Basic data, e.g. type of sample, sampling location, and creation date and time of the aliquot are mandatory. Identification of the person responsible for sample processing is stored with the sample or aliquot record. Container and storage location, including tower and rack (e.g. freezer, nitrogen tank, or breeding bench), are stored (see Fig. 1). For frozen samples, the amount of freezing cycles is notated to further define sample quality.

Samples and aliquots can be created one by one or by using the copy function for existing aliquots. For studies, large amounts of aliquots can be generated by applying study schemes (see above and Fig. 4).

Users can create any amount of storage systems with information on building, floor, room, and type of storage. Virtual boxes can be designed with a name and the box size, in which aliquots are placed. HEnRY will check for each position, if the slot is still free, to avoid doubled placements. A virtual look inside each box is possible (see Fig. 1), such that the user is at any time fully aware of the used space within the box and its content. Mass edit functions for almost all properties of the aliquot are available.

For the unique and secure identification of aliquots and samples, HEnRY provides the opportunity to print own customized labels. Labels can be created with or without QR code. The properties and their order printed on the label are user-customizable with a direct preview in the interface (see Fig. 5). Labels can be stored as templates and are accessible to every user in the database. After printing a label, the printer sign in the bio banking view turns from green to red to avoid double printing of aliquot labels (see Fig. 1).

### Shipment module

In the shipment module, aliquots are prepared for the shipment. Users can specify a shipment container and add aliquots for shipment to the box. It is possible to add aliquots to the virtual shipment box via scanning of the QR code or a search function using the patient ID. It is also possible to run full SQL statements from the interface for more complex searches. In cases where the storage box is identical to the shipment box, the whole box and the contained aliquots can be selected, by choosing the box. Reports concerning the content of a package content can be created and printed. The reports available in the shipment module contain a visual layout of the box and a list of aliquots with their properties (in the format. XPS or. CSV). The study supervisor can preselect the properties of the aliquots saved in the .csv file. A customization of the range of properties and their format is possible. In addition, a letter, to address the receiver of the shipment is printable.

Important information such as the reason and way of shipment as well as the delivery address can be given by the study assigned to the aliquots.

### User right administration

HEnRY features user management with different levels of access. The study supervisor has the right to manage and add studies within HEnRY. The study supervisor can monitor the completeness and the correctness of information contained in the HEnRY database. This guarantees ongoing and steady control of processing quality. After the end of a study, the study supervisor has the option to anonymize patient data in line with patient privacy rights. Laboratory staff is allowed to edit the sample processing protocol.

If HEnRY is linked to a clinical database, clinical and personal data of patients can be viewed by users with the “physician” right. Otherwise, only pseudonymous data is shown. Users in the “IT” user group can administrate the SQL connection, manage HEnRY users, and adjust printing settings.

### Export of aliquot data

Only pseudonymous data can be exchanged. Data of samples and aliquots can be exported into XML files [23], e.g. in order to merge data from one specific center with a central biobank. In our local use-case, merged data is monthly delivered to the LIMS of the DZIF [26].

### Queries and reports

HEnRY offers a variety of search functions. Aliquots can be selected by study, visit, storage or site.ne site is available. A graphical user interface offers a virtual view of a selected box, including the amount and placement of aliquots within the box. The monitoring status of all aliquots can be assessed and printed. All lists can be exported as XPS or CSV files.

Data can also be selected via an SQL query interface, and printed as scanable search lists with QR codes for each aliquot. By use of this tool, researchers can perform complex and customized searches covering all data contained in the database.

### HEnRY parser

To connect external data sources, a separate module is available for HEnRY, offering extract, transform, and load processes for different source formats (e.g. MDB, CSV, HL7). The parser is not part of the primary program package.

### Data protection and security

In view of the increasing requirements towards data protection standards in Europe and especially Germany, data privacy officers and clinical IT departments are sometimes reluctant to allow the use of LIMS tools based on cloud services and web interfaces, especially in the context of potentially stigmatizing diseases such as HIV. HEnRY can run with a locally administrated MSSQL database and offers hierarchical user rights on database and frontend levels. The storage of data, backup plans, and access to the data are fully controlled by the local IT of the hospital. Therefore, this tool can be seamlessly integrated into high security environments.

For large working groups, HEnRY works with a central MSSQL Server [21] and multiple clients. If access from outside the clinical network is necessary, HEnRY can be used via secure remote access solutions and has been successfully integrated into a Citrix Workspace environment [22].

## Results

In 2012, a survey was performed among HIV specialists and cohort researchers in the German Centre for Infection Research to identify the state of affairs and identify pressing needs in IT development across sites. Based on the survey results, a need for a harmonized collection and reporting of biomaterial samples was recognized. While some sites already had highly developed biobanks with professional software and storage solutions, most partners used legacy software products or standard spreadsheet software packages to maintain biomaterial and data collections. Based on the obvious demand for a cheap and efficient solution, the decision was made to develop a common tool for sample collection and exchange. A first list of desirable features was made and development of HEnRY started in 2014.

The lead developer of HEnRY went to on-site half-day visits to various users to directly understand the current and desired workflow and better address concerns and queries. Two working groups took the role of key users, who received early designs for commenting and took part in workshops between users and developers to identify core workflow parameters. Many key features of HEnRY such as study schemes have been developed in direct collaboration with the laboratory staff of the key user groups. During 11 meetings with both key users groups between 2015 and 2018, features were improved, performance optimized, and bugs removed. The current version is 2.2.

After successfully deploying productive versions of HEnRY for the key user groups in 2015 and 2016, HEnRY was requested by several other groups at UHC and DZIF. Table 1 shows an overview of the collected data for samples, aliquots and studies in HEnRY databases of different working groups at UHC. Currently, 2574 patients, 7953 samples, 68,519 aliquots are managed in HEnRY at the UHC for 23 ongoing studies.

**Table 1:** Overview over the data contained in different working groups using HEnRY; data from 10.01.2020

Laboratory	Research field	Installation date	Patients	Primary samples	Aliquots	Studies
**Group A**	Virology	Oct. 2015	1.086	2.459	34.229	10
**Group B**	Infectiology	Mar. 2016	291	1.203	20.499	1
**Group C**	Infectiology	May.2017	367	2.402	7.984	4
**Group D**	Infectiology	Aug. 2018	17	287	1.007	1
**Group E**	Dermatology	Nov. 2018	20	40	400	1
**Group F**	Nephrology	Jan. 2019	658	1.402	3.662	6
**Group G**	Virology	Sep. 2019	122	122	649	2
**Group H**	Tumorgentics with cell lines	Dez. 2019	3	18	15	0 (LIMS)
**Group I**	Oncology	Dez. 2019	10	20	74	4

To improve the accessibility of biomaterial within the scientific community, samples obtained with broad patient consent for certain biobanking studies are automatically reported to the central biomaterial registry (zentrale Bioprobenregister ZBR) of the TI Biobanking [26]. From there, samples can be selectively requested for research. As of now, 279 patient data sets were reported, along with 19,034 data sets for aliquots.

## Discussion

We performed a comprehensive comparison of HEnRY with other LIMS based on information available on the respective product websites [27–32]. Since many LIMS concentrate on the documentation of laboratory work and storage, only some advertise study workflow support [27, 29, 33–35] and laboratory process management [28] to promote multi-site studies. The possibility of creating customized labels was advertised by only few LIMS vendors [27, 28, 32]. The integration of shareable SQL queries for patient and sample identification was only identified in one other LIMS and linked to a specific licence [27]. As a free system, HEnRY is well suited for: (i) use in low resource environments, where HEnRY can be implemented without extensive IT experience on most Windows PCs with a free version of Microsoft SQL Server and (ii) multiple decentralized instances within one network, which is an asset especially for European research sites under the EU GDPR [36–45].

Our software is not without limitations. One shortcoming of HEnRY compared to commercial tools is the current lack of support for robotics and rack scanners [31]. However, since HEnRY is developed for scientific research groups, such features have not yet been requested by existing key users [46]. Another limitation of HEnRY is the C# code base, binding the product to the Microsoft software environment, which can be associated with higher costs for license fees for operating system and database servers. However, especially newly founded research groups may lack IT support to implement complex Linux and/or web-based solutions, but also funding for commercial systems and extensive external support. Given sufficient administrative rights, HEnRY can be setup with low to medium IT experience on a local Windows system and later be up-scaled for higher user numbers, connected systems, remote access, and ETL processes. Finally, HEnRY does not yet fulfil all desirable standards of semantic and syntactic interoperability. Still, with a comprehensive XML interface for sample data and workflows, package lists, and numerous ETL options for data import, interoperability in most environments can be achieved with moderate additional workload.

## Conclusion

With HEnRY, we provide a free versatile LIMS tool published open source under the MIT license and designed for coordination of local biobanks, as well as central management of multicentre studies.

The individual features of HEnRY, e.g. connecting sample and patient data, visualisation of boxes, sample tracking, and biorepository management, have already been offered through other biobanking systems in the past [27–32]. We are, however, unaware of any LIMS besides HEnRY that would combine all of these features in the context of an open source solution.

In conclusion, to our knowledge, HEnRY is the most comprehensive and versatile biobanking LIMS among the group of free and open source systems. HEnRY allows safe and rapid implementation of a local and/or central LIMS system, thus offering immense potential for emerging research groups, especially in the setting of limited resources and/or complex multicentre studies.

### Outlook

We envisage an extension of the list of annotatable sample and aliquot types, organisms and associated diseases, as well as machines used for sample processing, in order to improve the depictable level of documentation precision in non-HIV scientific research. Given the growing role of connectivity features and data exchange, standard meta-data and support for current health data exchange protocols such as HL7 FHIR [47] and MIABIS [48] are intended. With a sufficiently large user group, we intend transfer of copyright to a non-profit foundation for further development and maintenance of HEnRY.

## Supplementary information

**Additional file 1.** Supplementary material 1: Class Diagram HEnRY

**Additional file 2.** Supplementary material 2: Database scheme for aliquots and samples

**Additional file 3.** Supplementary material 3: Data flow 1

**Additional file 4.** Supplementary material 4: Data flow 2

**Additional file 5.** Supplementary material 5: Work flow

**Additional file 6.** Supplementary material 6: Use case with user rights

## Data Availability

The HEnRY software: can be downloaded from the tp-hiv.de website. source code is available on request. the printing function is optimized for the printer Labelident BP370i. The SQL Express Edition: can be downloaded from https://www.microsoft.com/de-DE/download/details.aspx?id=56840. to create a local database.

## Abbreviations

CAP: College of American Pathologist
DZIF: German Center for Infection Research
HEnRY: The bio banking tool HEnRY (HIV Engaged Research technologY)
HiObs: HIV Observation System; a software developed by the Robert Koch Institute
ISBER: International Society for Biological and Environmental Repositories
LIMS: A laboratory information management system
MIT: Massachusetts Institute of Technology
MSSQL: Microsoft SQL
RRH: Research Ready Hospital
RKI: Robert Koch Institute
SQL: Structured Query Language
TMF: Technology and method platform for networked medical research e.V
TopHIV study: Treatment of Primary HIV-1 Infection study
TP-HIV: Translational Platform HIV
TTU: Thematic Translational Unit HIV
WPF: Windows Presentation Foundation
ZBR: Central bio-sample register
