# Supplementary figures and images for: HEnRY: a DZIF LIMS tool for the collection and documentation of biomaterials in multicentre studies
Source: BMC Bioinformatics. 2020 Jul 8;21:290. doi: 10.1186/s12859-020-03596-1 (PMC7346399; doi:10.1186/s12859-020-03596-1)

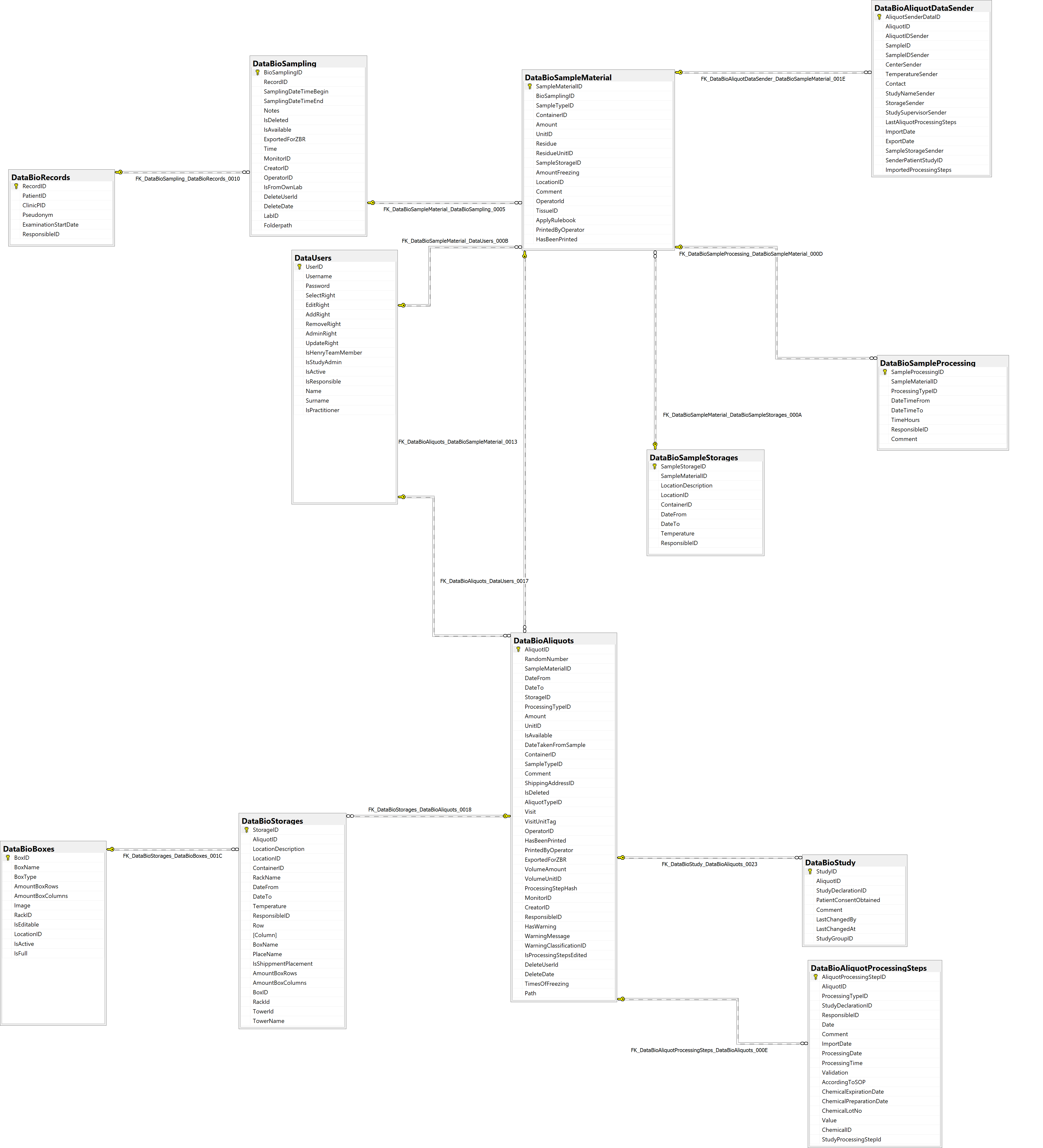

Supplement: Supplementary file 2 — Additional file 2. Supplementary material 2: Database scheme for aliquots and samples [file 12859_2020_3596_MOESM2_ESM.png]

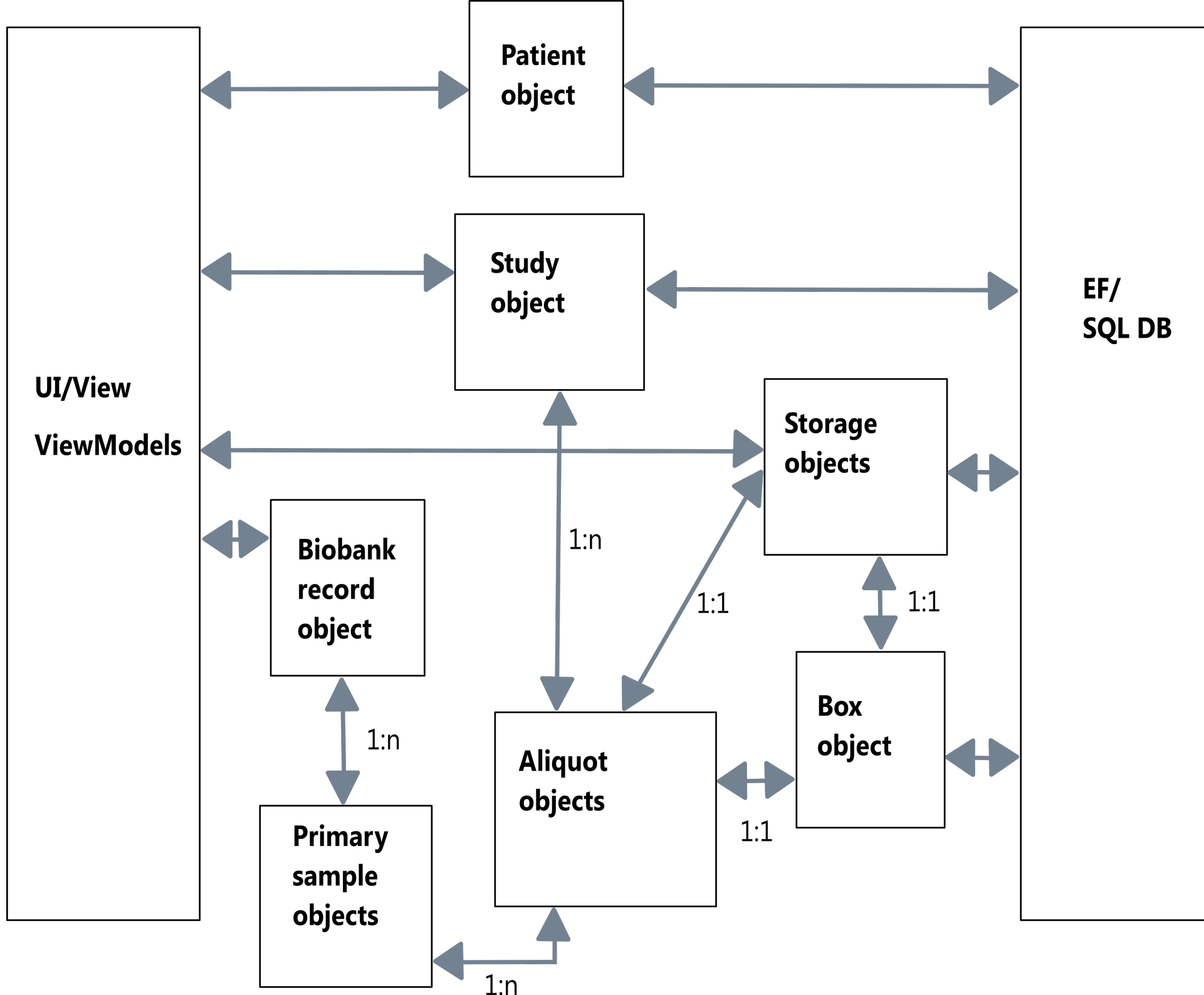

Supplement: Supplementary file 3 — Additional file 3. Supplementary material 3: Data flow 1 [file 12859_2020_3596_MOESM3_ESM.pdf]

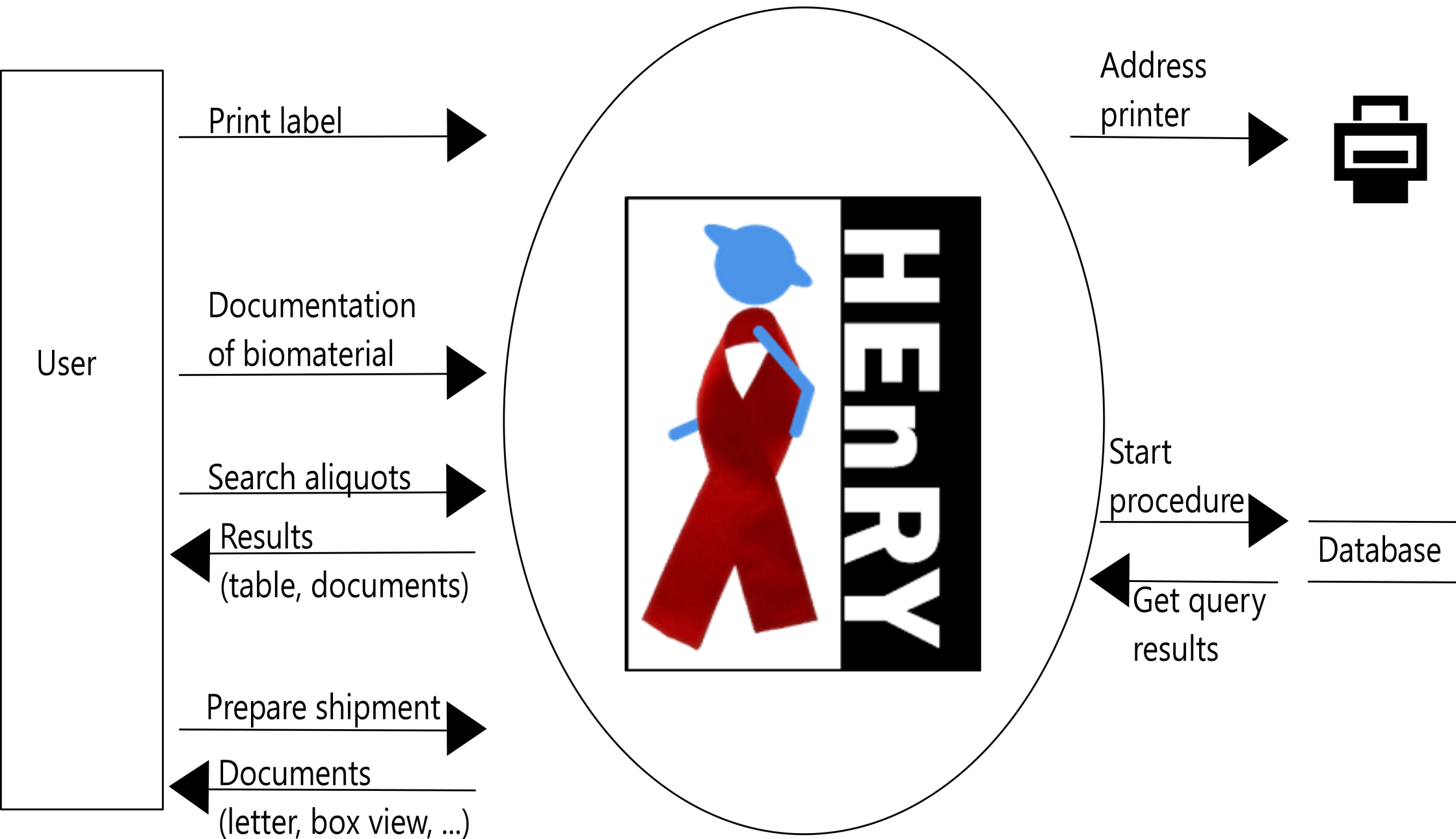

Supplement: Supplementary file 4 — Additional file 4. Supplementary material 4: Data flow 2 [file 12859_2020_3596_MOESM4_ESM.pdf]

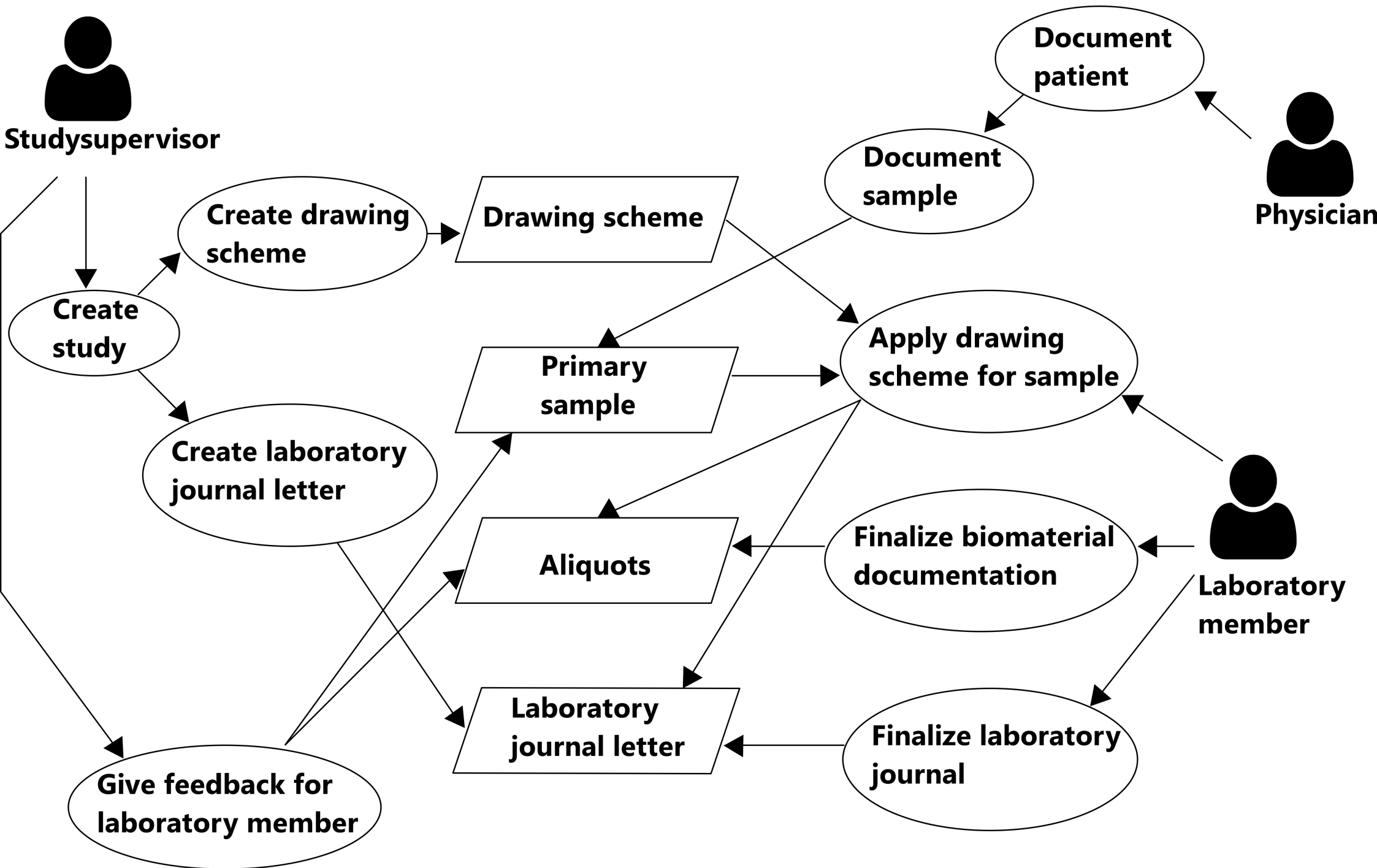

Supplement: Supplementary file 5 — Additional file 5. Supplementary material 5: Work flow [file 12859_2020_3596_MOESM5_ESM.pdf]

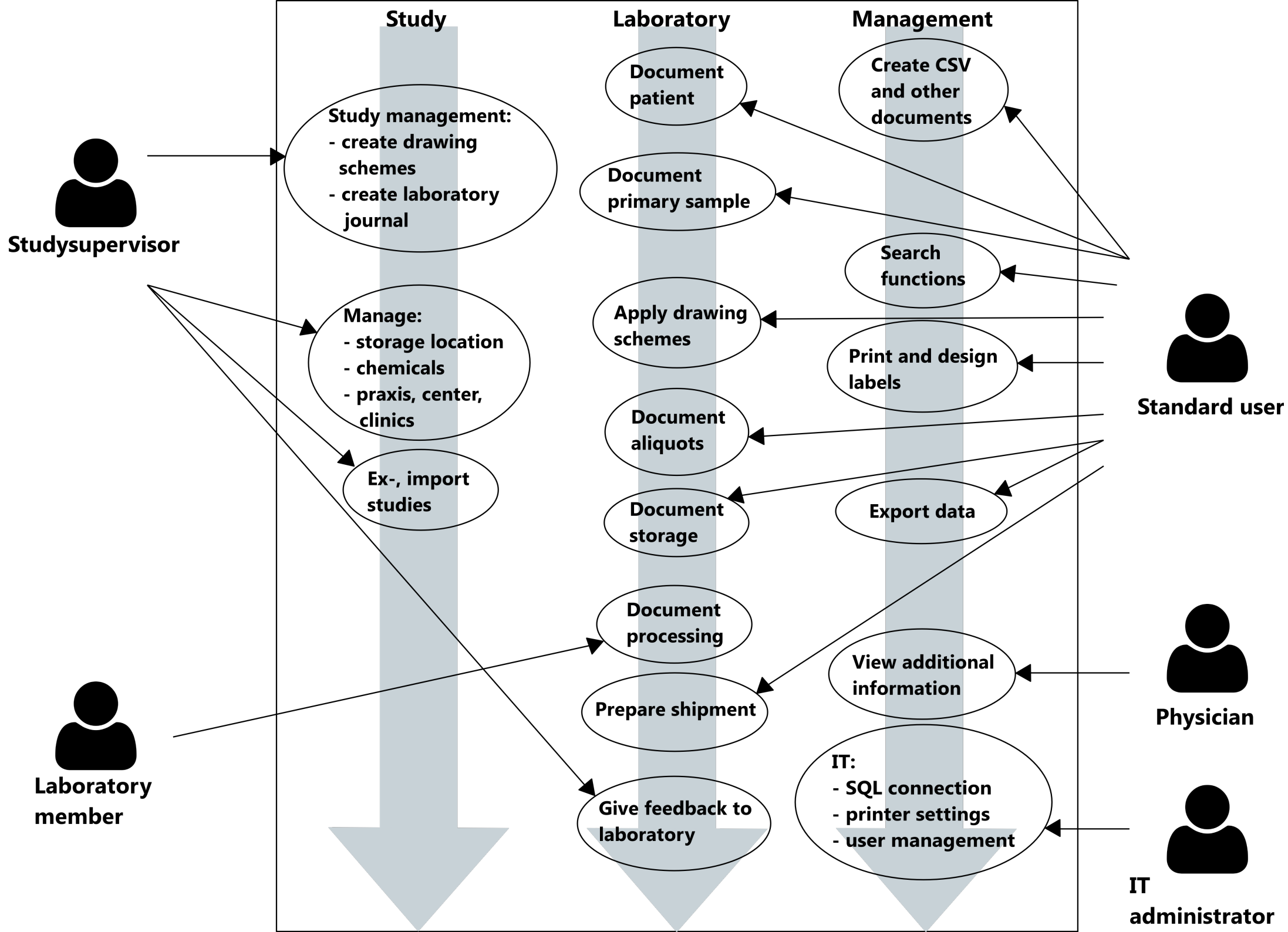

Supplement: Supplementary file 6 — Additional file 6. Supplementary material 6: Use case with user rights [file 12859_2020_3596_MOESM6_ESM.pdf]
